# Patient-reported outcomes, predictive modeling, and artificial intelligence in spine surgery: current limitations and future directions

**DOI:** 10.1007/s00590-026-05006-3

**Published:** 2026-11-05

**Authors:** Jacob F. Wood, Madhu Vishnu Sankar Reddy Rami Reddy, Shawn C. Murphy, Tarak Davuluri, Alastair Hoyt

**Affiliations:** 1 Department of Neurosurgery, Department of Surgery, The University of Toledo College of Medicine and Life Sciences, Toledo, USA; 2 Department of Neurosciences and Psychiatry, The University of Toledo College of Medicine and Life Sciences, Toledo, USA

**Keywords:** Artificial intelligence, Patient-reported outcomes, Predictive modeling, Spine surgery

## Abstract

**Introduction:**

Chronic back pain is a leading cause of disability worldwide, contributing to reduced productivity and diminished quality of life for many patients. Spine surgery, particularly lumbar decompression and fusion, serves as an intensive intervention after conservative measures fail. Despite advances in surgical technique and instrumentation, outcomes remain highly variable, with up to half of patients experiencing little or no meaningful improvement. This variability has prompted growing interest in predictive models that forecast which patients are most likely to benefit from surgery.

**Methods:**

This review examines the current landscape of predictive modeling for patient-reported outcomes (PROs) in spine surgery. We evaluated the use of PRO instruments, which have become central to assessing surgical success. We further explored existing predictive models that incorporate demographic, clinical, and psychosocial variables, as well as emerging applications of artificial intelligence (AI) and machine-learning techniques aimed at improving diagnostic accuracy and prognostic performance.

**Results:**

Current predictive models demonstrate variable performance across populations and remain constrained by incomplete datasets, inconsistencies in definitions, and limited integration of psychosocial factors. While most models report moderate discrimination for categorical outcomes (AUC 0.62–0.84), the subjective nature of PROs and the complexity of postoperative recovery pose challenges to accurate prediction. Machine-learning approaches, including neural networks, show promise in identifying complex relationships among diverse predictors of surgical outcomes. Future advancements will rely on leveraging multimodal data sources and integrating them through AI-driven frameworks. Transparency and external validation remain essential for clinical implementation.

**Conclusion:**

While PROs remain essential for understanding patient experiences and benchmarking surgical success, they often fall short as standalone predictive tools. The next generation of modeling should integrate multidimensional data and utilize AI’s capacity to detect clinically meaningful patterns. When implemented, these models have the potential to enhance preoperative counseling, guide surgical decision-making, and advance personalized spine care.

## Introduction

Chronic back pain represents a leading cause of disability worldwide and contributes to lost productivity and loss of quality of life for a patient [1, 2]. For many, spine surgery becomes a treatment of last resort after more conservative therapies, such as medications and physical therapy, fail [3, 4]. Procedures such as lumbar decompression and spinal fusion are among the most frequently performed musculoskeletal surgeries [5]. Despite surgical advances in orthopedics and neurological surgery, including minimally invasive approaches and cutting-edge instrumentation, outcomes for these procedures remain variable [6].

The main clinical question facing patients undergoing these procedures is simple: *how much improvement can they expect after surgery?* This uncertainty persists even in cases of technically flawless procedures [6]. Studies consistently demonstrate that a significant proportion of patients fail to achieve meaningful pain relief or functional improvement following surgery [7–9]. Depending on the cohort and outcome measures, as many as 40–50% of patients remain unchanged or experience their condition worsening following spine surgery [7–9].

Patient-reported outcomes (PROs) have become the primary means of evaluating spine surgery success, given that pain relief, a key determinant of surgical benefit, is inherently subjective [10–12]. It is important to distinguish between tools that measure outcomes (e.g., Oswestry Disability Index, Visual Analog Scale, Patient-Reported Outcomes Measurement Information System), which quantify patients’ pain and functional status, and predictive models, which use clinical, demographic, and psychosocial data to estimate the likelihood of improvement after surgery. Measurement tools are essential inputs for predictive models, but do not themselves forecast individual outcomes. Unlike fusion rates or radiographic decompression, which can be objectively measured, pain intensity and its impact on daily function are uniquely assessed through patient reporting [13]. This subjectivity, while important for capturing patient experience, presents a fundamental challenge for predictive models that attempt to forecast outcomes based on data that are not fully objective. PROs, often combined with thresholds such as the minimum clinically important difference (MCID), provide structured methods for evaluating whether patients report a meaningful benefit. However, they are not designed to predict outcomes at the individual level or to account for the full spectrum of factors influencing recovery [10–12, 14].

In response to these issues, predictive models have emerged as a potential solution. By incorporating clinical, demographic, and sometimes psychosocial variables, these models attempt to estimate which patients are most likely to experience improvement after surgery [15]. Their goal is to enhance patient selection, optimize counseling, reduce unnecessary operations, and allocate resources more effectively. Yet, despite enthusiasm and the use of advanced machine-learning techniques, predictive models have consistently underperformed in clinical practice [14, 15].

This review examines the limitations of current predictive models for PROs in spine surgery, explores the consequences of these imperfect tools, and considers future directions in modeling - particularly the potential of artificial intelligence (AI) to integrate diverse datasets and provide more accurate and clinically useful predictions.

## Patient reported outcomes in spine surgery

The measurement of surgical success often depends on the patient’s perception of their own outcome. Many factors contribute to this perception, including psychological attitude toward surgery and adherence to postoperative care, among other factors. Traditional metrics such as fusion rates or radiographic decompression often fail to capture the lived psychological or physical experience of pain and disability [16, 17]. Instead, PROs offer structured, standardized assessments of symptom burden, function, and quality of life [10, 17, 18].

Instruments such as the Oswestry Disability Index (ODI), Visual Analog Scale (VAS) for pain, and Patient-Reported Outcomes Measurement Information System (PROMIS) domains quantify patients’ perceptions of their health and function both before and after surgery [10, 19, 20]. The ODI is a widely used 10-item questionnaire assessing the functional disability in patients with back pain [19, 20]. The VAS is a simple, validated, and graphical scale that measures back and leg pain intensity on a numerical scale ranging from 1 to 10. PROMIS domains are computer-adaptive measures that assess physical, mental, and social health, including sub-categories such as alcohol use, anger, companionship, emotional support, and social isolation characteristics, among others [12, 21]. Some PRO tools are summarized in Table 1.

**Table 1:** Current PRO tools

Instrument	Primary domain assessed	Description/scale	Strengths	Limitations	Common MCID range
Oswestry Disability Index (ODI)	Functional disability	10-item questionnaire (0–100)	Widely used; validated	May not reflect psychosocial factors	9.5%–65.1% improvement
Visual Analog Scale (VAS)	Pain intensity	1–10 numeric or visual scale	Simple; patient-centered	Unidimensional; subjective	Varies by study
PROMIS Domains	Physical, mental, and social health	Computer-adaptive testing; multiple subdomains	Multidimensional; Multifactorial	Complex scoring; less intuitive	Not standardized across surgeries
SF-36	Health-related quality of life	36-item survey, 8 health domains	Comprehensive	Less specific to spine, subjective	4–5 points
Timed Up and Go (TUG)	Mobility/functional performance	Time to stand, walk, turn, return and sit	Objective, easy to perform	Non-spinal related influence	Time improvement, 2–3 s
Roland-Morris Disability Questionnaire (RMDQ)	Low back pain disability	24-item questionnaire	Simple	Subjective	3–5 points
JOA Myelopathy Evaluation	Cervical Myelopathy	17-point scale	Specific to myelopathy	Limited sensory specificity, scoring complexity	1–3 points (42% threshold minimum)
Neck Disability Index	Neck pain and disability	10-item questionnaire	Simple, widely used	Limited psychosocial assessment	7–10 points
Quebec Back Pain Disability Scale	Functional Disability	20-item scale	Spine specific	Less widely used	15–20% improvement
Spinal Stenosis Symptom Questionnaire (SSSQ)	Neurogenic Claudication Symptoms	Symptom severity and functional limitations	Spine specific	Less widely used	N/A

Summary of common patient-reported outcome tools used in spine surgery. This table outlines key characteristics of commonly used PRO instruments, including the primary domain assessed, scale description, notable strengths and limitations, and reported ranges for minimal clinically important difference (MCID) values

Predictive models use these tools to define meaningful improvement in surgical outcomes. Clinicians frequently apply the MCID, defined as the smallest change in a score perceived as beneficial by the patient [22]. MCID thresholds vary by instrument, measurement, and surgery type; however, across studies, approximately 9.5%- 65.1% of patients achieve MCID after surgery, leaving anywhere from 34.9% − 90.5% of patients without clinical benefit [1, 23–25].

While PROs and associated data are crucial for research and benchmarking, they have limitations. First, heterogeneity in outcome definitions (ODI vs. VAS vs. PROMIS) complicates comparison across studies [10, 11, 14]. Second, reliance on MCID introduces variability, as thresholds are not universally standardized [1, 24, 25]. Third, PROs are inherently subjective - shaped by psychological state, social context, and expectations - underscoring the challenge of using PROs as both outcomes and predictive targets.

## The promise of predictive models

The variability in outcomes following spine surgery has led to growing interest in predictive modeling as a tool to enhance clinical decision-making. Unlike traditional outcome measures, which assess patient progress only after surgical intervention, predictive models seek to identify the likelihood of achieving meaningful improvement before surgery. This shift from retrospective evaluation to prospective prediction holds clear appeal for both clinicians and patients.

By incorporating demographic factors (e.g., age, sex, body mass index), clinical variables (e.g., baseline pain intensity, comorbidities, prior surgeries), and psychosocial determinants (e.g., depression, anxiety, social support), predictive models aim to generate individualized estimates of surgical success [14]. In practice, this can help surgeons stratify risk, set realistic expectations, and guide patients toward or away from operative management. From a systems perspective, predictive models also aim to reduce unnecessary procedures, optimize healthcare expenditures, and direct resources toward patients most likely to benefit.

## Building better predictive models

Improving predictive accuracy in spine surgery will require expanding beyond traditional clinical metrics to include psychosocial, socioeconomic, and mental health factors. A previous study has demonstrated that cognitive appraisal variables accounted for nearly 40% of the variance in spine surgery outcomes after adjustment for demographic and clinical factors, further supporting the need to incorporate cognitive and psychosocial domains into predictive models [26]. Evidence suggests that depression, anxiety, catastrophizing, resilience, and social support exert strong influence on recovery trajectories, yet these variables are rarely captured in current datasets [27]. Similarly, previous studies showed that socioeconomic status, insurance coverage, employment, and housing stability may shape both access to care and postoperative outcomes. Preoperative narcotic or opioid use is another important predictor that should be specifically captured, as it may reflect chronic pain severity, central sensitization, opioid tolerance, psychological distress, and greater baseline disability, all of which can influence postoperative pain perception and satisfaction.

However, a large retrospective cohort study found that although patients from poorer communities present with worse baseline disability and pain, community-level socioeconomic indices poorly predict improvement [28, 29]. Although large registries and electronic health records may increasingly capture socioeconomic variables, they often lack direct measures of cognitive appraisal, expectations, coping style, pain catastrophizing, resilience, and patient-specific interpretation of disability. Incorporating these domains into large-scale registries, alongside baseline pain perception and coping strategies, will allow models to better reflect the complex, real-world determinants of recovery.

Recent advances in computational methods have expanded the possibilities of such modeling. Regression-based clinical prediction rules laid the foundation, but newer approaches increasingly utilize machine-learning algorithms capable of capturing complex, nonlinear interactions among variables [30]. A previous study developed and validated machine-learning models for predicting disability and pain after lumbar disc surgery, demonstrating strong discrimination (C-statistics 0.74–0.81) and superiority over traditional regression-based prediction tools [31]. Random-forest algorithms have achieved accuracies up to 87.6% in perioperative complication prediction, while neural networks excel in modeling individualized surgical outcomes and three-dimensional anatomical reconstructions (Table 2). Support vector machines and optimized k-nearest-neighbor (KNN) with oversampling (SMOTE) techniques have also improved prediction accuracy, reaching approximately 76% with large datasets (Table 2) [30, 32]. The hope is that these methods can reveal subtle data patterns that traditional statistical models overlook, ultimately producing more personalized and actionable predictions.

**Table 2:** Current predictive models

Predictive model	Population / surgery type	Outcome predicted	Key predictors	Modeling approach	Performance (AUC / Accuracy)
Spine Patient Outcomes Research Trial (SPORT) predictive model	Lumbar disc surgery	Functional improvement (ODI)	Age, baseline ODI, comorbidities, symptom duration	Logistic regression	AUC 0.63–0.72
Norwegian registry ML Models	Lumbar disc herniation	Treatment success (ODI and pain scores)	Baseline ODI, prior duration, anxiety, depression	Various (e.g. XGBoost, RF)	AUC > 0.81(disability), > 0.74 (pain)
KNN + SMOTE model for perioperative complications	Mixed lumbar surgeries	Complication risk	Age, lab values, surgery type, socioeconomic status	K-nearest neighbors with oversampling SMOTE	Accuracy 76%, F1- score 67%
Random forest model for post-laminectomy outcomes	Lumbar decompression	PRO improvement	Baseline ODI, VAS, psychosocial scores	Random forest	Accuracy ~ 87.6%

Summary of common predictive models that are currently utilized in spine surgery. This table outlines the predictive model, the population the model is utilized on, the outcome predicted, key predictors for modeling, the approach of modeling, and performance accuracy based on aggregate studies.

Differences in predictive performance across models likely reflect differences in patient populations, surgical indications, outcome definitions, follow-up intervals, feature availability, and analytic strategy. For example, regression-based models such as SPORT-derived tools are generally more interpretable but may be limited in their ability to capture nonlinear interactions. In contrast, machine-learning approaches such as random forests, gradient boosting, support vector machines, and neural networks may better model complex relationships among baseline disability, psychosocial factors, comorbidities, and perioperative variables. However, these methods may be less transparent, particularly when model predictions are generated through “black-box” architectures without clear explanation of which variables drive risk estimates.

Beyond clinical utility, predictive models may also strengthen clinical research infrastructure. Large registries and electronic health record datasets provide opportunities for external validation and iterative model improvement across diverse patient populations. Emerging predictive frameworks increasingly incorporate novel data modalities, including advanced imaging analytics, wearable-sensor metrics, and patient-generated health data, to improve model granularity and translational relevance. Recent systematic and conceptual reviews emphasize that smart-wearable devices, Internet-of-Things (IoT) sensors, and connected implantable devices generate continuous biomechanical and physiological data that can refine predictive algorithms for spinal recovery, surgical outcomes, and complication risks [18, 33, 34].

Furthermore, digital twin technology combined with advanced AI represents a new frontier in personalized spine surgery and outcome optimization. A digital twin is a detailed virtual replica of a patient’s spine, constructed from CT or MRI imaging and often enhanced by AI-driven segmentation to generate accurate three-dimensional anatomical reconstructions [35]. These models integrate patient-specific anatomical, biomechanical, and sensor-based movement data, enabling simulations that mirror each patient’s unique characteristics [33, 35]. Finite element analysis (FEA) can then be applied to predict how the spine responds to mechanical forces such as bending, loading, or implant placement, supporting optimization of implant positioning and surgical strategy [33]. Advanced AI platforms allow real-time “what-if” simulations, incorporating physics-informed neural networks (PINNs) and continuous sensor monitoring to inform both intraoperative decisions and post-operative rehabilitation plans [36]. Digital twins can rapidly estimate tissue properties and predict clinical outcomes with high accuracy, and likely even with greater precision as AI evolves [37]. By reducing surgical errors and personalizing postoperative care, digital twin technology has the potential to complement predictive modeling and AI-driven frameworks, ultimately advancing precision spine surgery. These tools could transform preoperative counseling and shared decision-making by providing patients with tailored forecasts of pain relief and functional recovery. However, applying the vast data from these models into consistent, clinically meaningful improvements in practice remains challenging.

Importantly, biomechanical fidelity should not be conflated with PRO predictability. A patient-specific model may improve understanding of spinal mechanics, loading behavior, and implant positioning, but technically successful fusion or accurate instrumentation does not necessarily translate into pain relief, satisfaction, or functional improvement. Therefore, digital twins and FEA should be viewed as complementary tools within a broader AI-enabled framework rather than standalone solutions to the psychosocial and cognitive limitations of current PRO prediction models.

Future predictive models must also leverage multimodal data streams. Natural language processing can extract psychosocial context from unstructured clinical notes, while imaging features from radiographs, MRI, or CT scans may capture structural correlates of pain not visible in standard documentation. Biological and genetic markers could shed light on pain susceptibility, and wearable sensors can provide continuous, real-time data on mobility and activity. AI methods, particularly machine learning and deep learning, are uniquely suited to integrate these high-dimensional data sources, identifying nonlinear interactions and subtle predictors that elude conventional models. Neural networks can fuse imaging with clinical data, gradient boosting algorithms can handle missing data and complex interactions within noisy EMR databases, and natural language processing can transform narrative records into quantifiable features [38–40].

For these advances to translate into clinical benefit, transparency and interpretability will be essential. This is particularly important for black-box AI systems, which may generate accurate predictions without clearly explaining whether risk is driven by modifiable psychosocial factors, baseline disability, opioid use, comorbidities, imaging findings, or surgical complexity. In spine surgery, interpretability is essential because predictions must be translated into interventions, such as expectation-setting, mental health optimization, opioid reduction, prehabilitation, or reconsideration of operative candidates. Surgeons and patients alike must understand not only the predictions themselves but also the reasoning behind them. Approaches such as importance rankings and visual explanation tools can clarify model reasoning, helping to build trust in high-stakes decisions. Ultimately, these systems should function as decision-support tools that augment rather than replace a surgeon’s judgment. By enhancing shared decision-making and aligning expectations, AI-powered predictive models may evolve into patient-centered guides that improve both counseling and surgical outcomes.

Despite promising retrospective and registry-based performance, many predictive models for spine surgery outcomes remain in development, validation, or limited institutional implementation rather than routine prospective clinical deployment. As a result, there is limited evidence that model-guided decision-making has consistently changed rates of surgery, reoperation, postoperative resource utilization, or long-term PRO trajectories. Future studies should prospectively evaluate whether these tools improve counseling, patient selection, prehabilitation referral, opioid optimization, psychological support, and postoperative follow-up intensity.

## The future role of PROs in AI-based prediction

An important consideration is whether PRO instruments will retain their current role as predictive modeling becomes increasingly driven by machine learning and multimodal data. PROs and predictive models should not be considered competing approaches. Instruments such as the ODI, VAS, and PROMIS were primarily developed to quantify patient-perceived disability, pain, and health status rather than independently forecast postoperative outcomes [41–43]. Accordingly, their greatest future value may be as both clinically meaningful outcome targets and structured inputs within broader predictive frameworks. Baseline PRO scores can capture dimensions of disability and symptom burden that may not be represented by demographic, radiographic, or perioperative variables, while machine-learning models can evaluate these measures in combination with psychosocial, clinical, imaging, and longitudinal data.

Available evidence suggests that increasingly complex models can achieve improved discrimination in selected populations; however, higher retrospective model performance does not necessarily establish clinical superiority [18, 44–46]. Differences in patient populations, outcome definitions, follow-up intervals, predictor availability, and validation strategies may inhibit direct comparison among existing studies. Furthermore, relatively little evidence demonstrates that adding PRO data to a predictive model improves immediate or long-term outcomes compared with otherwise similar models without PRO inputs. Prospective studies should therefore evaluate the incremental predictive value of individual PRO instruments and determine whether their contribution changes across short- and long-term postoperative endpoints.

It also remains unclear whether currently available PRO instruments are equally suitable for machine-learning applications. ODI, VAS, PROMIS, and other instruments differ substantially in dimensionality, scoring structure, disease specificity, psychosocial content, and longitudinal responsiveness [41, 42]. These instruments were generally developed and validated as clinical measurement tools rather than optimized features for predictive algorithms. Consequently, the PRO that is most useful for measuring postoperative improvement may not necessarily provide the greatest predictive information before surgery [18, 41–43].

Future development of PRO instruments may therefore require an additional consideration: predictive utility. Rather than evaluating questionnaires solely according to reliability, validity, responsiveness, and MCID, future studies could examine their incremental contribution to model discrimination, calibration, generalizability, and longitudinal prediction. Machine-learning methods may also identify individual questionnaire items or combinations of domains that contain greater predictive information than conventional composite scores. This could ultimately support development of shorter, adaptive, or prediction-oriented instruments designed to retain patient-centered meaning while providing high-value inputs for AI-based models. Such an approach would not replace traditional validation of PROs but rather extend their role from standalone measurement instruments toward components of integrated, patient-centered predictive systems.

## Conclusion

Despite advances in surgical technique and outcome measurement, spine surgery continues to yield variable results, with many patients failing to achieve meaningful benefit. Patient-reported outcomes provide essential insight into the lived experiences of pain and function but remain limited as predictive tools. Current predictive models, though promising, are constrained by incomplete data, heterogeneity in outcome definitions, and limited generalizability. Emerging applications of AI and multimodal data integration hold significant potential to refine prognostic accuracy. However, the success of AI models will depend on transparency, mitigation of bias, and seamless incorporation into clinical workflows. Predictive models should function as decision-support systems that complement surgeon judgment, enhance shared decision-making, and advance patient-centered care, while AI models continue to mature and improve over time.

## Data Availability

No datasets were generated or analysed during the current study.
